# Efficacy and safety of antiangiogenic agents or chemotherapy plus EGFR‐TKIs in advanced non‐small cell lung cancer: A systematic review and network meta‐analysis

**DOI:** 10.1111/1759-7714.14783

**Published:** 2023-01-02

**Authors:** Jiali Dai, Xinyin Liu, Jun Li, Tianyu Qu, Yanan Cui, Shidai Jin, Erbao Zhang, Renhua Guo

**Affiliations:** ^1^ Department of Oncology The First Affiliated Hospital of Nanjing Medical University Nanjing China; ^2^ Department of Epidemiology Center for Global Health, School of Public Health, Nanjing Medical University Nanjing China; ^3^ Jiangsu Key Lab of Cancer Biomarkers, Prevention and Treatment, Collaborative Innovation Center for Cancer Personalized Medicine Nanjing Medical University Nanjing China

**Keywords:** antiangiogenic agents, chemotherapy, epidermal growth factor receptor inhibitors, network meta‐analysis, non‐small cell lung cancer

## Abstract

**Background:**

The combination of antiangiogenic agents with epidermal growth factor receptor inhibitors (EGFR‐TKIs) and chemotherapy with EGFR‐TKIs are the most common combination treatment options in epidermal growth factor receptor (EGFR) positive non‐small cell lung cancer (NSCLC). This network meta‐analysis was performed to evaluate the differences between them.

**Methods:**

We searched the PubMed, EMBASE and the Cochrane Controlled Trials Register up to August 2022. The primary outcomes were progression‐free survival (PFS) and objective response rate (ORR). The secondary endpoints were overall survival (OS), disease control rate (DCR) and adverse events (AEs). The data of hazard ratio (HR) or risk ratio (RR) with their corresponding 95% confidence intervals (CIs) were extracted in the studies. A network meta‐analysis (NMA) was used to indirectly compare the efficacy and safety of antiangiogenic agents plus EGFR‐TKIs and chemotherapy plus EGFR‐TKIs.

**Results:**

Pooled data of included studies were demonstrated that chemotherapy plus EGFR‐TKIs had a benefit in ORR compared to antiangiogenic agents plus EGFR‐TKIs in patients with *EGFR* mutated NSCLC (RR = 1.1, 95% CI: 1.0–1.2). However, there were no significant differences in PFS, OS and DCR between in the two group (PFS: HR = 1.0, 95% CI: 0.74–1.6; OS: HR = 0.78, 95% CI: 0.45–1.5; DCR: RR = 1.0, 95% CI: 0.94–1.1). The common treatment‐related AEs in the two groups were relatively manageable.

**Conclusion:**

Based on the efficacy and safety, the combination of chemotherapy with EGFR‐TKIs is considered the best combination treatment options in advanced NSCLC with *EGFR* mutation.

## Graphical abstract

Novel findings: Growing evidence demonstrates that EGFR‐TKIs combined with other therapies can significantly alleviate drug resistance and improve the therapeutic effect. For patients with advanced EGFR+ NSCLC, antiangiogenic therapy and chemotherapy are the most common combination therapeutic regimens. Vascular endothelial growth factor inhibits angiogenesis by binding to the vascular endothelial growth factor receptor, which activates proangiogenic signaling. The dual inhibition of the EGFR and VEGF pathway has been proved to significantly enhance antitumor activity in vivo and in vitro. Moreover, the combination of chemotherapy and EGFR‐TKIs provides synergistic antitumor activity by activating extracellular signal‐regulated kinases and promoting apoptosis which may overcome acquired resistance to chemotherapy. Whether antiangiogenic agents plus EGFR‐TKIs or chemotherapy plus EGFR‐TKIs deliver different clinical outcomes remains unknown. Neither option has been studied head‐to‐head in a randomized controlled trial (RCT) to compare the effectiveness and safe. A network meta‐analysis (NMA) was conducted to indirectly compare antiangiogenic therapy plus EGFR‐TKIs versus chemotherapy plus EGFR‐TKIs in advanced *EGFR*‐mutated NSCLC. Based on the findings, we were interested in identifying the best choice in advanced EGFR positive NSCLC.

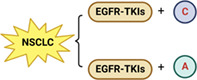

## INTRODUCTION

Lung cancer is one of the most common malignant tumors and the leading cause of cancer‐related mortality in the world.^1^ Nearly 85% of these cases are due to non‐small cell lung cancer (NSCLC).^2^ At diagnosis, the majority of patients are already at advanced stages, leading to a poor prognosis. In western countries, 11%–16% of NSCLC patients have epidermal growth factor receptor (*EGFR*) mutation, whereas 50% are Asian.^3^ Numerous clinical trials have suggested that EGFR tyrosine kinase inhibitors (TKIs) improve progression‐free survival (PFS) and the objective response rate (ORR).^4^, ^5^, ^6^, ^7^ EGFR‐TKIs are regarded as standard therapies in patients with EGFR‐positive (EGFR+) NSCLC. Unfortunately, acquired resistance still occurs in patients after receiving EGFR‐TKIs, leading to tumor recurrence and metastasis.

Growing evidence demonstrates that EGFR‐TKIs combined with other therapies can significantly alleviate drug resistance and improve the therapeutic effect.^7^, ^8^, ^9^, ^10^, ^11^, ^12^, ^13^, ^14^, ^15^, ^16^, ^17^, ^18^, ^19^, ^20^, ^21^, ^22^, ^23^, ^24^, ^25^, ^26^ For patients with advanced EGFR+ NSCLC, antiangiogenic therapy and chemotherapy are the most common combination treatment options. Vascular endothelial growth factor (VEGF) inhibits angiogenesis by binding to the vascular endothelial growth factor receptor, which activates proangiogenic signaling.^27^, ^28^ Among the most common angiogenesis modulators used in the treatment of advanced NSCLC are bevacizumab and ramucirumab. The dual inhibition of the EGFR and VEGF pathway has been proved to significantly enhance antitumor activity in vivo and in vitro.^15^, ^18^, ^24^ Moreover, adding a cytotoxic chemotherapy, such as pemetrexed, carboplatin or paclitaxel, to EGFR‐TKIs showed clinical improvement in patients with NSCLC.^5^, ^7^, ^17^ The combination of chemotherapy and EGFR‐TKIs provides synergistic antitumor activity by activating extracellular signal‐regulated kinases and promoting apoptosis which may overcome acquired resistance to chemotherapy.^23^ Whether antiangiogenic agents plus EGFR‐TKIs or chemotherapy plus EGFR‐TKIs deliver different clinical outcomes remains unknown. To the best of our knowledge, there have been no head‐to‐head randomized controlled trials (RCTs) to compare the effectiveness and safety.

A network meta‐analysis (NMA) was conducted to indirectly compare antiangiogenic therapy plus EGFR‐TKIs versus chemotherapy plus EGFR‐TKIs in advanced *EGFR*‐mutated NSCLC. Based on the findings, we were interested in identifying the best choice in advanced EGFR positive NSCLC.

## METHODS

### Search strategy

Our search was conducted using the databases PubMed, EMBASE, and Cochrane Controlled Trials Register for the period up to August 2022. Additionally, we analyzed abstracts from the meetings of the American Society of Clinical Oncology (ASCO) and the European Society of Medical Oncology (ESMO). The keywords used included “epidermal growth factor receptor inhibitors, EGFR‐TKIs, antiangiogenic agents, bevacizumab, ramucirumab, chemotherapy, non‐small cell lung cancer and NSCLC”. The NMA followed the Preferred Reporting Items for Systematic Reviews and Meta‐analysis (PRISMA) guidelines and the PRISMA extension statement for NMAs.^29^

### Selection criteria

The inclusion criteria of the NMA included the following: (i) the patients had advanced *EGFR*‐mutated NSCLC; (ii) the study was an RCT or prospective cohort study; (iii) the treatment arm was antiangiogenic agents plus EGFR‐TKIs or chemotherapy plus EGFR‐TKIs, and the control arm was EGFR‐TKIs alone; (iv) the study reported efficacy outcomes or adverse events (AEs). The following studies were excluded: (i) the patients did not receive antiangiogenic agents plus EGFR‐TKIs or chemotherapy plus EGFR‐TKIs; (ii) no *EGFR* mutations were identified; and (iii) the study was a retrospective study, review, case report, meta‐analysis, or non‐English publication.

### Data extraction and quality assessment

Separately, two investigators extracted the structured data from the included trials. The third reviewer clarified any disagreements and doubts. Data were summarized as follows: first author, publication year, study design, ethnicity, treatments, number of patients, age, sex, smoking status, histological type, stage, *EGFR* mutation, outcome and toxicity. The quality assessment of the included studies was evaluated using RevMan software (version 5.3).

### Statistical analysis

Primary outcomes of this study were PFS and ORR, with secondary outcomes being OS, disease control rate (DCR) and adverse events. Hazard ratios (HR) and 95% CIs of the outcome measures (OS, PFS) were taken directly from the original studies or indirectly from the survival curves. The relevant effect measure of ORR and DCR were the risk ratio (RR) with 95% CI. For toxicity, the outcome measure of grade ≥3 AEs was the RR with 95% CI.

Direct meta‐analysis was performed using STATA version 14.0 software (Stata Corporation, College Station, Texas, USA). For indirect comparisons, the analysis of network meta‐data was performed in R (version 3.6.1) and using R packages. The *I* ^*2*^ and *x* ^*2*^ tests were used to evaluate heterogeneity. *p* < 0.1 or *I* ^*2*^ > 50% denoted significant heterogeneity, in which case the random‐effects model was applied to evaluate the data. Other than that, the fixed‐effects model was employed. *p* < 0.05 indicated statistical significance.

## RESULTS

### Characteristics of included trials

**FIGURE 1:**
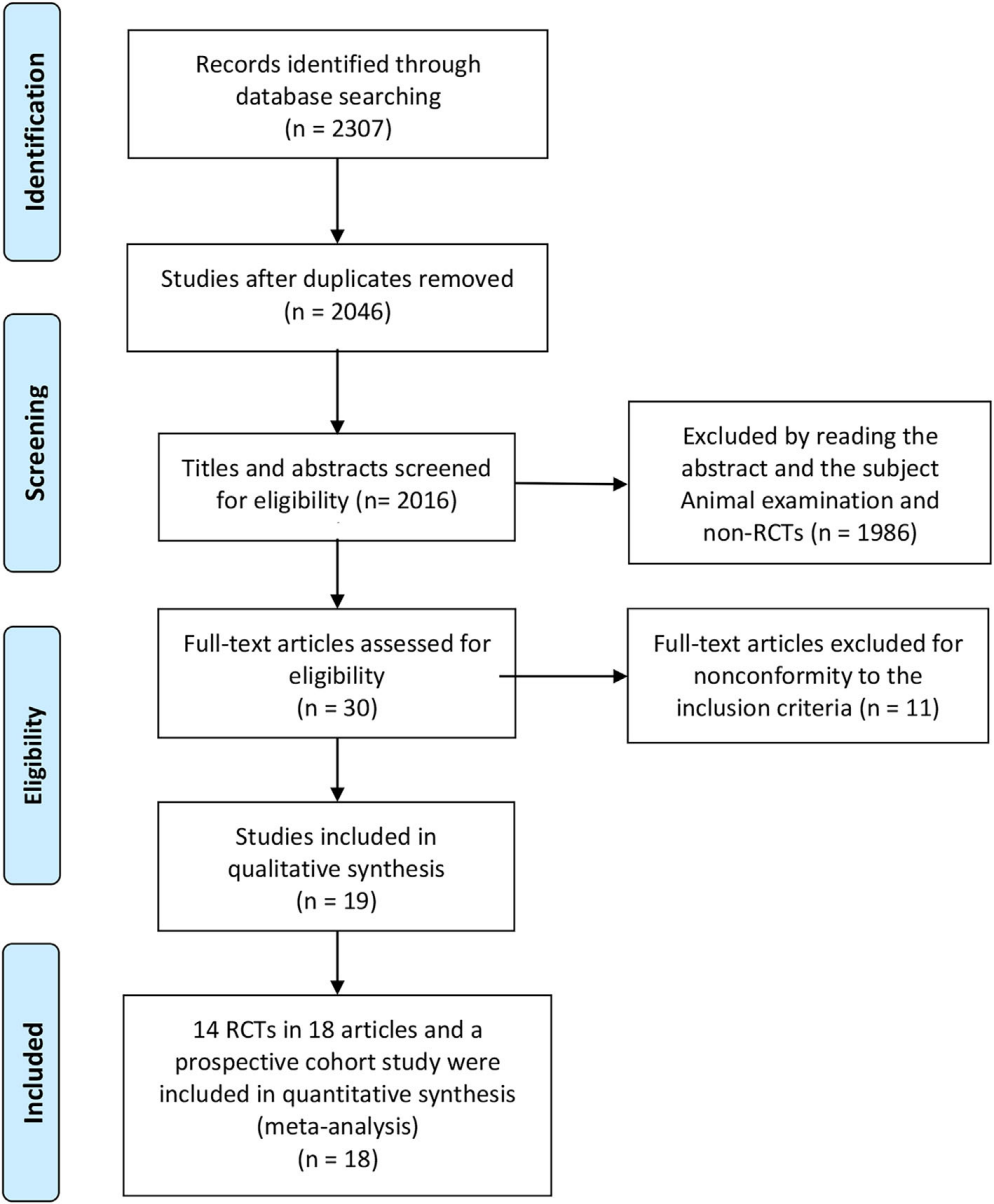
Flowchart for search results and selection details. RTC, randomised controlled trial

**FIGURE 2:**
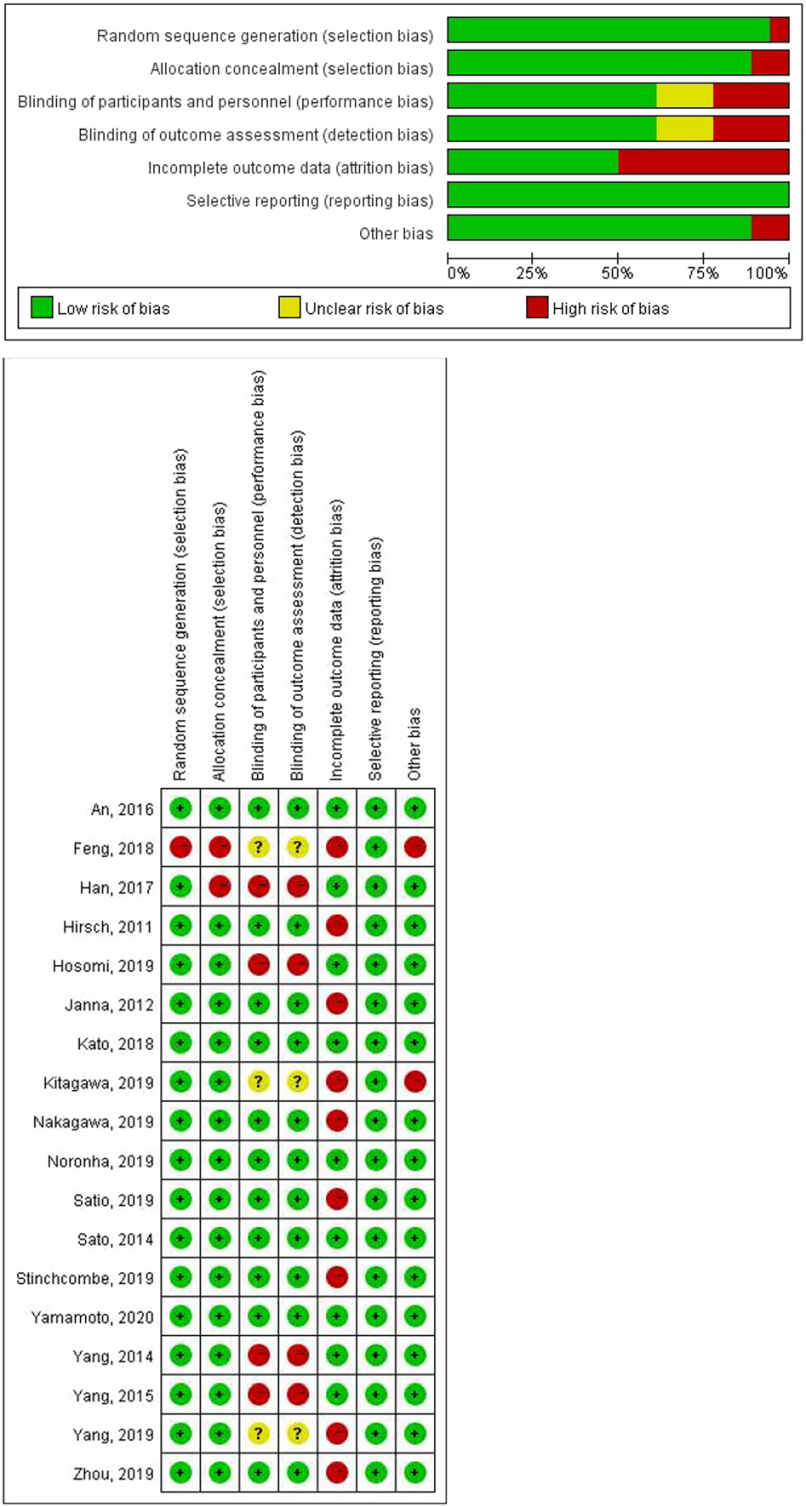
Risk of bias graph for all studies included

The flow diagram of the study was listed according to PRISMA guidelines (Figure 1). Fourteen RCTs reported in 19 articles and one prospective cohort study were included in the NMA. In total, 1185 EGFR‐positive patients were identified from the 19 articles. A summary of the baseline characteristics of included studies is in Table 1. As a control, EGFR‐TKI alone or EGFR‐TKI plus placebo were used. The treatment arm was antiangiogenic agents (bevacizumab/ramucirumab) in nine articles. The treatment group of the other 10 studies was chemotherapy. The majority of patients enrolled in our NMA were Asian. In three trials, the ethnicities were European or other. In order to assess the research quality, we used the Cochrane Risk of Bias Tool (Figure 2).

**TABLE 1:** Characteristics of studies included in the meta‐analysis

Authors	Year	Study design	Ethnicity	Treatment	Sample size	Age, year (median)	Sex (male; female)	Smoking status (never smoker; smoker; other)	ECOG score (0; 1)	Histological type (adeno carcinoma; large cell carcinoma; squamous cell carcinoma; other)	Stage (IIIb; IV; recurrence)	EGFR mutation (19del; L858R)	Outcome
Seto et al.	2014	RCT	Asian	BEV + ERL	75	67	30; 45	42; 9; 24	43; 32	74; 0; 0; 1	1; 60; 14	40; 35; 0	PFS; ORR; DCR;AEs
				ERL	77	67	26; 51	45; 6; 26	41; 36	76; 1; 0; 0	0; 62; 15	40; 37; 0	
Kato et al.	2018	RCT	Asian	BEV + ERL	75	67	30; 45	42; 9; 24	43; 32	74; 0; 0; 0	1; 60; 14	40; 35; 0	AEs
				ERL	77	67	26; 51	45; 6; 26	41; 36	76; 1; 0; 0	0; 62; 15	40; 37; 0	
Feng et al.	2018	Prospective cohort study	Asian	BEV + EGFR TKI	25	62	7; 18	22; 3; 0	NA	25; 0; 0; 0	0; 25; 0	10; 15; 0	PFS; OS; ORR; DCR
				EGFR TKI	30	68	13; 17	24; 6; 0	NA	30; 0; 0; 0	0; 30; 0	11; 19; 0	
Kitagawa et al.	2019	RCT	Asian	BEV + GEF	6	73.5	1; 5	4; 2; 0	2; 4	6; 0; 0; 0	0; 6; 0	4; 2; 0	PFS; ORR; DCR;AEs
				GEF	10	72.5	3; 7	8; 2; 0	7; 3	9; 0; 0; 1	0; 9; 1	6; 3; 1	
Nakagawa et al.	2019	RCT	Asian, 77%	RAM + ERL	224	65	83; 141	134; 64; 26	116; 108	215; 0; 0; 9	0; 195; 29	123; 99; 2	PFS; ORR; DCR;AEs
				ERL	225	64	83; 142	139; 73; 16	119; 106	218; 0; 0; 7	0; 189; 36	120; 105; 0	
Saito et al.	2019	RCT	Asian	BEV + ERL	112	67	41; 71	65; 6; 41	64; 48	110; 1; 0; 1	8; 82; 22	56; 56; 0	PFS; ORR; DCR;AEs
				ERL	112	68	39; 73	64; 7; 41	68; 42	112; 0; 0; 0	8; 84; 20	55; 57; 0	
Stinchcombe et al.	2019	RCT	White, 85%	BEV + ERL	43	65	12; 31	25; 17; 1	24; 19	NA, nonsquamous	0; 39; 4	29; 14; 0	PFS, OS, ORR, AEs
				ERL	45	63	14; 31	23; 22; 0	19; 26	NA, nonsquamous	0; 39; 6	30; 15; 0	
Yamamoto et al.	2021	RCT	Asian	BEV + ERL	75	67	30; 45	42; 33; 0	43; 32	74; 0; 0; 1	1; 60; 14	40; 35; 0	PFS, OS
				ERL	77	67	26; 51	45; 32; 0	41; 36	76; 1; 0; 0	0; 62; 15	40; 37; 0	
Zhou et al.	2021	RCT	Asian	BEV + ERL	157	57	60; 97	NA	25; 132	157; 0; 0; 0	4; 141; 12	82; 75; 0	PFS, ORR, AEs
				ERL	154	59	58; 96	NA	17; 137	154; 0; 0; 0	6; 134; 14	79; 75; 0	
Hirsch et al.	2011	RCT	European	PAC + CAR + ERL	6	NA	NA	NA	NA	NA	NA	NA	PFS; OS; ORR; DCR
				ERL	9	NA	NA	NA	NA	NA	NA	NA	
Jänne et al.	2012	RCT	Multinational	PAC + CAR + ERL	33	NA	NA	NA	NA	NA	NA	NA	PFS; OS; ORR
				ERL	33	NA	NA	NA	NA	NA	NA	NA	
Yang et al.	2014	RCT	Asian	PEM + CIS + GEF	26	NA	NA	NA	NA	NA	NA	NA	PFS; ORR; DCR; AEs
				GEF	24	NA	NA	NA	NA	NA	NA	NA	
Yang et al.	2016	RCT	Asian	PEM + CIS + GEF	26	NA	NA	NA	NA	NA	NA	NA	OS
				GEF	24	NA	NA	NA	NA	NA	NA	NA	
An et al.	2016	RCT	Asian	PEM + GEF	45	65.72	25; 20	20; 25; 0	NA	45; 0; 0; 0	4; 41; 0	16; 29; 0	PFS; OS; ORR; DCR; AEs
				GEF	45	66.89	25; 20	19; 26; 0	NA	45; 0; 0; 0	6; 39; 0	17; 28; 0	
Cheng et al.	2016	RCT	Asian	PEM + GEF	126	62	44; 82	81; 45	39; 87	NA, nonsquamous	0; 105; 21	65; 52; 9	PFS; ORR; DCR; AEs
				GEF	65	62	24; 41	47; 18	21; 44	NA, nonsquamous	0; 57; 8	40; 23; 2	
Han et al.	2017	RCT	Asian	PEM + CAR + GEF	40	NA	15; 25	27; 13; 0	8; 32	40; 0; 0; 0	8; 32; 0	21; 19; 0	PFS; OS; ORR; DCR; AEs
				GEF	41	NA	18; 23	27; 14; 0	9; 32	41; 0; 0; 0	5; 36; 0	21; 20; 0	
Yang et al.	2020	RCT	Asian	PEM + GEF	126	62	44; 82	81; 45	39; 87	NA, nonsquamous	0; 105; 21	65; 52; 9	PFS; OS; AEs
				GEF	65	62	24; 41	47; 18	21; 44	NA, nonsquamous	0; 57; 8	40; 23; 2	
Noronha et al.	2020	RCT	Asian	PEM + CAR + GEF	174	54	88; 86	145; 29; 0	1; 137	170; 0; 1; 3	3; 171; 0	107; 60; 7	PFS; OS; ORR; DCR; AEs
				GEF	176	56	93; 83	150; 26; 0	7; 130	170; 0; 1; 5	5; 171; 0	109; 60; 7	
Hosomi et al.	2020	RCT	Asian	PEM + CAR + GEF	170	64.8	56; 114	96; 73; 0	98; 72	168; 0; 0; 2	6; 139; 24	93; 69; 8	PFS; OS; ORR; DCR; AEs
				GEF	172	64	64; 108	97; 75; 0	107; 65	170; 0; 0; 2	4; 137; 30	95; 67; 10	

Abbreviations: AEs, adverse events; BEV, bevacizumab; CAR, carboplatin, cisplatin; DCR, disease control rate; EGFR TKI, epidermal growth factor receptor inhibitor; ERL, erlotinib; GEF, gefitinib; NA, not available; ORR, overall response rate; OS, overall survival; PAC, paclitaxel; PEM, pemetrexed; PFS, progression‐free survival; RAM, ramucirumab; RCT, randomized controlled trial.

### Direct comparisons

As shown in Figure S1, antiangiogenic agents combined with EGFR‐TKIs were the treatment group in nine of the studies. In advanced NSCLC patients with *EGFR* mutation, combining antiangiogenic agents with EGFR‐TKIs significantly increased the PFS compared to treatment with EGFR‐TKIs alone (HR = 0.60, 95% CI: 0.52–0.69, *p* < 0.001) (Figure S1A, Table S1). In terms of OS, ORR and DCR, the two arms did not differ significantly (OS: HR = 0.91, 95% CI: 0.73–1.13, *p* = 0.405; ORR: RR = 1.04, 95% CI: 0.98–1.10, *p* = 0.232; DCR: RR = 1.01, 95% CI: 0.98–1.03, *p* = 0.606) (Figure S1B–D, Table S1).

In 10 studies, EGFR‐TKIs were used alone or in combination with chemotherapy. Chemotherapy and EGFR‐TKI treatment resulted in significantly longer PFS in patients with EGFR‐positive NSCLC (HR = 0.68, 95% CI: 0.51–0.89, *p* = 0.005) (Figure S2A, Table S1). For ORR, EGFR‐TKI treatment added to chemotherapy was superior to the EGFR‐TKI therapy alone, as shown in Figure S2B (RR = 1.17, 95% CI: 1.09–1.25, *p* < 0.001) (Figure S2B, Table S1). However, there was no indication that chemotherapy combined with EGFR‐TKIs was superior in OS and DCR over EGFR‐TKIs alone (OS: HR = 0.74, 95% CI: 0.53–1.02, *p* = 0.068; DCR: RR = 1.02, 95% CI: 0.97–1.07, *p* = 0.363) (Figure S2C‐D, Table S1).

### Network comparisons

**FIGURE 3:**
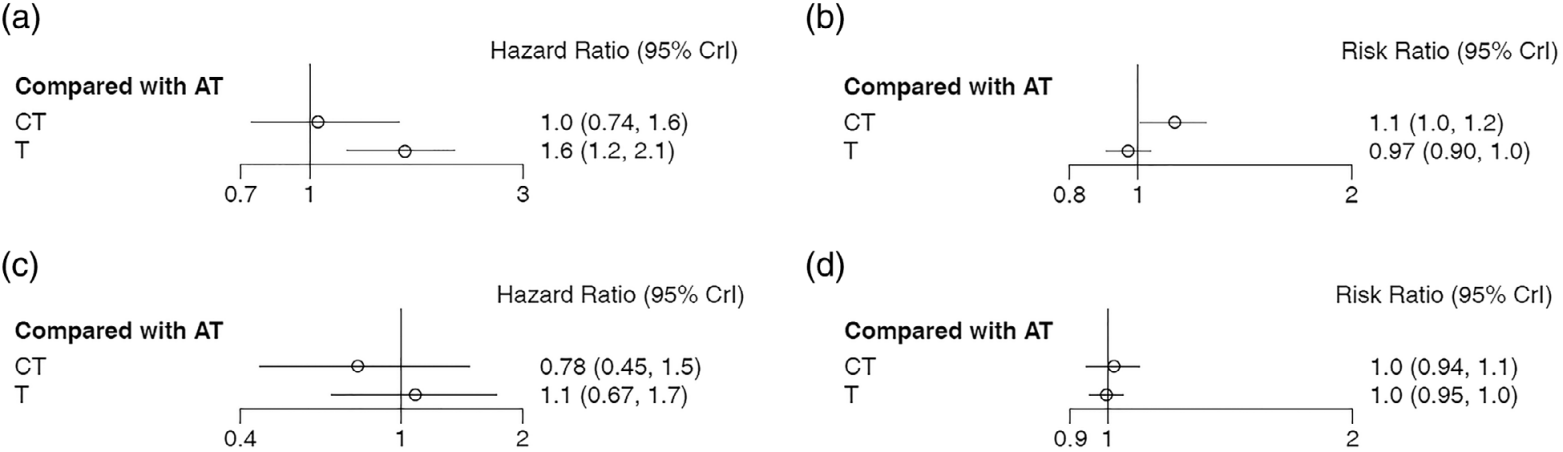
Forest plots of the (a) progression‐free survival, (b) overall survival, (c) objective response rate and (d) disease control rate that are associated with antiangiogenic agents plus epidermal growth factor receptor inhibitors (EGFR‐TKIs) versus chemotherapy plus EGFR‐TKIs from network meta‐analysis comparisons. AT, antiangiogenic agents plus epidermal growth factor receptor inhibitor

In our NMA, chemotherapy plus EGFR‐TKIs constituted the treatment group; antiangiogenic agents and EGFR‐TKIs were regarded as the control group. All 15 trials in 19 articles presented available PFS and ORR information. No marked benefit in PFS was seen from the combination of antiangiogenic agents plus EGFR‐TKIs over chemotherapy and EGFR‐TKIs (HR = 1.0, 95% CI: 0.74–1.6) (Figure 3a, Table 2). For ORR, a response benefit was seen with chemotherapy plus EGFR‐TKIs compared to antiangiogenic agents plus EGFR‐TKIs (RR = 1.1, 95% CI: 1.0–1.2) (Figure 3b, Table 2).

**TABLE 2:** Network meta‐analysis results of treatment comparisons

Outcomes	HR/RR	95% CIs
PFS, HR (95%CI)	1.0	(0.74, 1.6)
Exon 19 del	0.87	(0.57, 1.4)
Exon 21 L858R	0.96	(0.57, 1.6)
OS, HR (95% CI)	0.78	(0.45, 1.5)
ORR, RR (95% CI)	**1.1**	**(1.0, 1.2)**
DCR, RR (95% CI)	1.0	(0.94, 1.1)

Abbreviations: AT, antiangiogenic agents plus epidermal growth factor receptor inhibitor; CIs, 95% confidence intervals; CT, chemotherapy plus epidermal growth factor receptor inhibitors; DCR, disease control rate; Exon 19 del, EGFR exon 19 deletion; Exon 21 L858R, EGFR exon 21 p.Leu858Arg; HR, hazard ratio; ORR, overall response rate; OS, overall survival; PFS, progression‐free survival; RR, risk ratio; T, epidermal growth factor receptor inhibitor.

The bold part represents the specific statistical significance, and its *p* value is less than 0.05.

All 11 studies published the findings of OS and DCR. There were no significant statistical differences between the two groups in OS (HR = 0.78, 95% CI: 0.45–1.5) (Figure 3c, Table 2). As a result of our network meta‐analysis, there were no significant differences between the two arms based on DCR data (RR = 1.0, 95% CI: 0.94–1.1) (Figure 3d, Table 2).

### Adverse events

As shown in Table 3, treatment‐related AEs were reported in 14 articles. As a general rule, diarrhea and rash were the most common grade ≥3 adverse events associated with EGFR TKI treatment. Diarrhea and rash were not significantly different between the two combination treatments (diarrhea: RR = 0.47, 95% CI: 0.089–1.7; rash: RR = 0.77, 95% CI: 0.36–1.5) (Table 3). Hypertension and proteinuria were substantially increased in patients treated with angiogenesis inhibitors and EGFR‐TKIs (hypertension: RR = 4.88, 95% CI: 3.49–6.81, *p* < 0.001; proteinuria: RR = 12.83, 95% CI: 3.98–41.38, *p* < 0.001) (Table 3). EGFR‐TKIs combined with chemotherapy had the highest risk of hematological toxicities, including anemia, leukopenia, neutropenia and thrombocytopenia (anemia: RR = 4.94, 95% CI: 2.91–8.39, *p* < 0.001; leukopenia: RR = 6.91, 95% CI: 3.12–15.33, *p* < 0.001; neutropenia: RR = 10.53, 95% CI: 6.19–17.93, *p* < 0.001; thrombocytopenia: RR = 28, 95% CI: 5.62–139.46, *p* < 0.001) (Table 3).

**TABLE 3:** Network meta‐analysis results of grade ≥3 adverse events comparisons

AEs	AT vs. T	CT vs. T	CT vs. AT
Diarrhea, RR (95% CI)	**2.9 (1.1, 10.0)**	1.4 (0.49, 3.2)	0.47 (0.089, 1.7)
Rash, RR (95% CI)	**1.3 (0.87, 1.8)**	0.96 (0.51, 1.8)	0.77 (0.36, 1.5)
Hypertension, RR (95% CI)	**4.88 (3.49, 6.81)**	‐	‐
Proteinuria, RR (95% CI)	**12.83 (3.98, 41.38)**	‐	‐
Anemia, RR (95% CI)	‐	**4.94 (2.91, 8.39)**	‐
Leukopenia, RR (95% CI)	‐	**6.91 (3.12, 15.33)**	‐
Neutropenia, RR (95% CI)	‐	**10.53 (6.19, 17.93)**	‐
Thrombocytopenia, RR (95% CI)	‐	**28.00 (5.62, 139.46)**	‐

Abbreviations: AT, antiangiogenic agents plus epidermal growth factor receptor inhibitor; CT, chemotherapy plus epidermal growth factor receptor inhibitors; T, epidermal growth factor receptor inhibitor; RR, risk ratio; CIs, 95% confidence intervals.

The bold part represents the specific statistical significance, and its *p* value is less than 0.05.

### Subgroup analysis

A subgroup analysis was performed based on seven trials reporting data on PFS based on *EGFR* mutation type. A comparison of PFS did not reveal obvious differences in the chemotherapy plus EGFR‐TKI group versus the antiangiogenic agents plus EGFR‐TKI group in patients with either EGFR exon 19 deletion or exon 21 L858R mutation (exon 19 del: HR = 0.87, 95% CI: 0.57–1.4; exon 21 L858R: HR = 0.96, 95% CI: 0.57–1.6) (Table 2).

## DISCUSSION

With the advancement of molecular biology and sequencing technology, some NSCLC patients have been found to have *EGFR*‐activating mutations, especially in the Asian population.^3^ The overall survival of NSCLC patients harboring *EGFR* mutations is significantly prolonged by EGFR‐TKIs.^4^, ^5^, ^6^, ^7^ EGFR‐TKIs have been regarded as the most effective regimen in patients with *EGFR* mutations. Unfortunately, acquired resistance still develops in patients after EGFR‐TKI treatment, leading to tumor recurrence and metastasis. Recently, some studies have shown that combining EGFR‐TKIs with other antitumor drugs is one strategy to overcome acquired resistance.^23^, ^24^

Over the past several decades, platinum‐based chemotherapy regimens have been used most frequently in treating advanced NSCLC. These patients commonly undergo chemotherapy regimens with platinum drugs coupled with pemetrexed and paclitaxel. As a result of a growing number of studies on tumor angiogenesis, antiangiogenic agents have been found to possess superior benefits for advanced NSCLC patients.^27^, ^28^ Treatment with EGFR‐TKIs in conjunction with chemotherapy and antiangiogenic agents are most common combination treatments in advanced EGFR+ NSCLC. Numerous clinical trials have demonstrated a significant survival benefit from combinations of antiangiogenic agents and EGFR‐TKIs versus EGFR‐TKIs alone.^8^, ^9^, ^10^, ^11^, ^12^, ^13^, ^14^, ^15^, ^26^ Chemotherapy combined with EGFR‐TKIs has been shown to be superior to EGFR‐TKIs alone in terms of PFS and ORR.^7^, ^16^, ^17^, ^18^, ^19^, ^20^, ^21^, ^22^, ^24^, ^25^ There are no RCTs that have directly compared chemotherapy plus EGFR‐TKIs with antiangiogenic agents plus EGFR‐TKIs. As far as we know, this is the first network meta‐analysis examining the efficacy and safety of combined treatment regimens in patients with advanced NSCLC who have an *EGFR* mutation.

As shown in our network meta‐analysis, chemotherapy plus EGFR‐TKIs gave an obvious benefit over antiangiogenic agents plus EGFR‐TKIs in terms of ORR, while there was no significant difference between the two groups in OS, PFS and DCR. In addition, deletions of exon 19 and L858 mutations of exon 21 led to similar survival benefits in the chemotherapy plus EGFR‐TKI group as well as using antiangiogenic agents plus EGFR‐TKI. Chemotherapy plus EGFR‐TKI was associated with a higher percentage of grade ≥3 hematological toxicities than antiangiogenic agent plus EGFR‐TKI. Nevertheless, the combination of antiangiogenic agents with EGFR‐TKIs had the highest risk of grade ≥3 hypertension and proteinuria. Among the EGFR‐TKI alone group, diarrhea and rash were the most common symptoms. The incidence of grade ≥3 diarrhea and rash associated with EGFR‐TKIs did not increase significantly in the groups receiving combined treatment. According to the findings of this network meta‐analysis, the combination of chemotherapy plus EGFR‐TKIs was the most effective combination treatment option for patients with EGFR‐positive advanced NSCLC.

Our network meta‐analysis is limited by several factors. First, the clinical data are reliant on indirect comparisons of the results from different trials rather than direct comparisons. Regarding efficacy and toxicity, the standards of each study were not completely consistent. In addition, we lack the original clinical data and cannot conduct in‐depth analysis. Second, the sample sizes in some included trials were relatively small, so their results need further validation. Third, some included RCTs lacked complete survival time data, which may affect the final analysis. Direct head‐to‐head RCTs are needed to evaluate the differences in efficacy and safety of chemotherapy and antiangiogenic agents along with EGFR‐TKIs in the future.

In conclusion, based on the results of the network meta‐analysis, chemotherapy plus EGFR‐TKIs produced an increase in ORR compared with antiangiogenic agents and EGFR‐TKIs alone. Nevertheless, the differences in OS, PFS and DCR between the two arms were not statistically significant. The common treatment‐related AEs were relatively manageable. Chemotherapy coupled with EGFR‐TKIs is considered the best combination treatment option for patients with advanced NSCLC expressing EGFR.

## AUTHOR CONTRIBUTIONS

Jiali Dai, Renhua Guo, Erbao Zhang and Shidai Jin conceived and designed the studies. Jiali Dai, Xinyin Liu, Jun Li, Tianyu Qu and Yanan Cui obtained the data of experiments. Jiali Dai, Xinyin Liu and Jun Li wrote the manuscript. All authors approved the final manuscript.

## CONFLICT OF INTEREST

No conflict of interest is declared by the authors.

## Supporting information


**Data S1:** Supporting Information

## Data Availability

In response to requests from the corresponding author, data supporting these findings can be accessed.
