# Supplementary material for: Efficacy and safety of antiangiogenic agents or chemotherapy plus EGFR‐TKIs in advanced non‐small cell lung cancer: A systematic review and network meta‐analysis
Source: Thorac Cancer. 2023 Jan 2;14(6):535–43. doi: 10.1111/1759-7714.14783 (PMC9968601; doi:10.1111/1759-7714.14783)
Supplement: Supplementary file 1 — Data S1: Supporting Information [file TCA-14-535-s001.pdf]

Supplemental Information

Figure S1

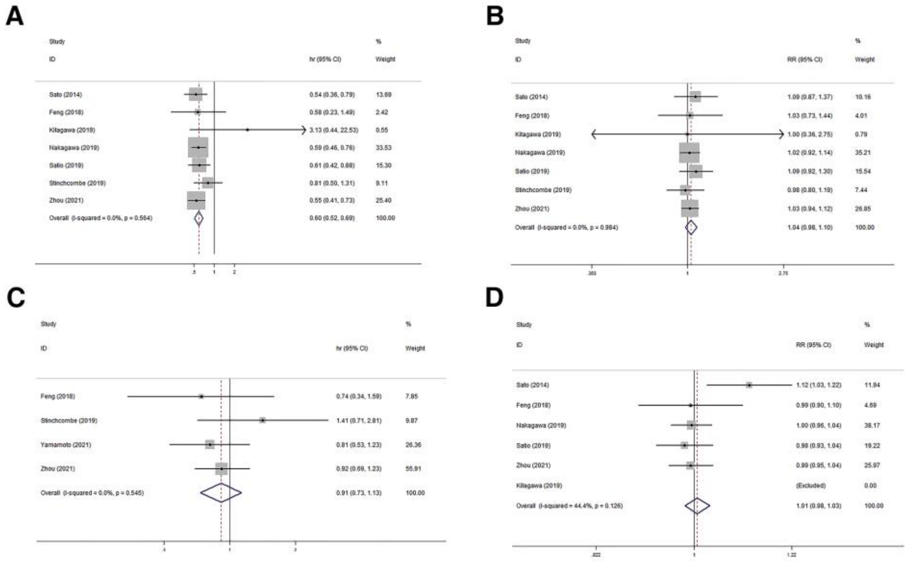

Figure S1. Forest plots of the (A) progression-free survival, (B) overall survival, (C) objective response rate, (D) disease control rate that are associated with antiangiogenic agents plus EGFR-TKIs vs. EGFR-TKIs.

Figure S2

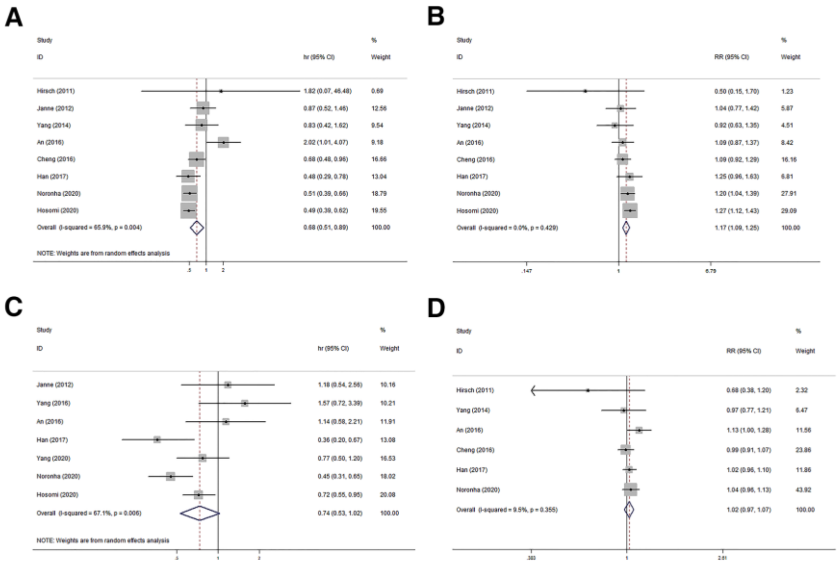

Figure S2. Forest plots of the (A) progression-free survival, (B) overall survival, (C) objective response rate, (D) disease control rate that are associated with chemotherapy plus EGFR-TKIs vs. EGFR-TKIs.

Table S1 Direct comparisons results of treatment comparisons.

	AT vs T	CT VS T
PFS, HR (95%CI)	**0.60 (0.52, 0.69)**	**0.68 (0.51, 0.89)**
OS, HR (95%CI)	0.91 (0.73, 1.13)	0.74 (0.53, 1.02)
ORR, RR (95%CI)	1.04 (0.98, 1.10)	**1.17 (1.09, 1.25)**
DCR, RR (95%CI)	1.01 (0.98, 1.03)	1.02 (0.97, 1.07)

Abbreviations: PFS: progression-free survival; OS: overall survival; ORR: overall response rate; DCR: disease control rate; AT: antiangiogenic agents plus epidermal growth factor receptor inhibitor; CT: chemotherapy plus epidermal growth factor receptor inhibitors; T: epidermal growth factor receptor inhibitor; HR: hazard ratio; RR: risk ratio; CIs: 95% confidence intervals.
